# Feasibility and Efficacy of Prolonged Exposure for PTSD among Individuals with a Psychotic Spectrum Disorder

**DOI:** 10.3389/fpsyg.2017.00977

**Published:** 2017-06-28

**Authors:** Anouk L. Grubaugh, Kimberly Veronee, Charles Ellis, Wilson Brown, Rebecca G. Knapp

**Affiliations:** ^1^Ralph H. Johnson Veterans Affairs Medical Center, CharlestonSC, United States; ^2^Department of Psychiatry and Behavioral Sciences, Medical University of South Carolina, CharlestonSC, United States; ^3^Department of Communication Sciences and Disorders, East Carolina University, GreenvilleNC, United States; ^4^Department of Public Health Sciences, Medical University of South Carolina, CharlestonSC, United States

**Keywords:** severe mental illness (SMI), trauma, exposure therapy, psychotic disorder, schizophrenia

## Abstract

**Objective:** Few empirical studies have examined the feasibility of trauma-focused treatment among individuals with schizophrenia. This lack of research is important given the substantial overlap of trauma exposure and subsequent PTSD with psychotic spectrum disorders, and the potential for PTSD to complicate the course and prognosis of schizophrenia and other variants of severe mental illness.

**Method:** As part of a larger study, 14 veterans with a psychotic spectrum disorder were enrolled to receive prolonged exposure (PE) for PTSD within a single arm open trial study design. Patient reactions and responses to PE were examined using feasibility indices such as attrition, survey reactions, and treatment expectancy; pre and post-changes in PTSD severity and diagnostic status; and thematic interviews conducted post-intervention.

**Results:** Quantitative and qualitative data indicate that implementation of PE is feasible, subjectively well-tolerated, and may result in clinically significant reductions in PTSD symptoms in patients with psychotic spectrum disorders.

**Conclusion:** Consistent with treatment outcome data in clinical populations with a broader range of severe mental illnesses, the current results support the use of PTSD exposure-based interventions, such as PE, for individuals with psychotic spectrum disorders.

## Introduction

It is well documented that individuals with severe and persistent forms of mental illness (SMI) are at increased risk for the experience of a traumatic event and subsequent development of post-traumatic stress disorder (PTSD; see Grubaugh et al., 2011 for review). Specifically, current and lifetime prevalence rates of PTSD in this population range between 46 and 53% (Grubaugh et al., 2011). Additionally, a comorbid diagnosis of PTSD among individuals with SMI is highly correlated with decreased psychosocial functioning, worse quality of life, and substance use (e.g., Mueser et al., 2004a,b; Ford and Fournier, 2007; Fan et al., 2008; Grubaugh et al., 2011); as well as a range of other prognostic indicators such as homelessness, disability ratings, suicidal ideation (e.g., Sautter et al., 1999; Mueser et al., 2004b; Strauss et al., 2006; Newman et al., 2010), and exacerbations in the core symptoms of SMI diagnoses (e.g., Kilcommons and Morrison, 2005; Lysaker et al., 2005; Schenkel et al., 2005; Üçok and Bikmaz, 2007; Lysaker and Larocco, 2008; Meade et al., 2009).

Despite elevated risk of PTSD and the associated impairment among individuals with SMI, the literature regarding the implementation of trauma- and PTSD-focused psychotherapy in this clinical population remains underdeveloped. This gap in data is due in part to the historical exclusion of patients with current psychotic symptoms, recent histories of suicidal or unstable behavior, and/or severe illness burden from participation in PTSD clinical trials (Spinazzola et al., 2005); as well as concerns by some clinicians that intense trauma focused interventions may be ‘over-stimulating’ for individuals with SMI (Braiterman, 2004; Fowler, 2004; Frueh et al., 2006a). Published studies include a handful of small open trials (Rosenberg et al., 2004; Mueser et al., 2007; Lu et al., 2009) and one randomized controlled trial (RCT), each of which used a similar cognitive restructuring intervention in samples of community mental health center (CMHC) patients (Mueser et al., 2008). Two additional open trials examined the efficacy of an exposure-based intervention for PTSD among individuals with SMI, one in a CMHC and the other in a VA Medical Center (Frueh et al., 2009; Grubaugh et al., 2016, respectively). Finally, one small RCT and two larger RCTs conducted in the Netherlands with community outpatients reported on the efficacy of two common types of exposure therapy (de Bont et al., 2013, 2016; van den Berg et al., 2015). Collectively, results from these trials suggest that specialized PTSD interventions are effective among patients with SMI (both civilian and veteran) and result in statistically significant reductions in PTSD symptoms across treatment (Frueh et al., 2009; Grubaugh et al., 2016) or between active and control group conditions (Mueser et al., 2008).

Although preliminary PTSD treatment outcome data for individuals with SMI are promising, there is significant room for an increased understanding of how this subset of trauma survivors respond to targeted PTSD intervention—particularly individuals with a psychotic spectrum disorder, given diathesis stress models of psychosis (Grubaugh et al., 2011). As such, the current manuscript used mixed qualitative/quantitative methods to better understand how a sample of 14 patients with a psychotic spectrum disorder (i.e., schizophrenia, schizoaffective, and psychotic disorder NOS) responded to Prolonged Exposure (PE) for PTSD (Foa and Rothbaum, 1998) using a range of feasibility indices, post-treatment thematic interview data, and symptom severity measures.

## Materials and Methods

### Overview of Study

The current study was part of a larger open trial of PE for PTSD among veterans with a psychotic spectrum disorder (Grubaugh et al., 2016). The active intervention phase (i.e., treatment phase) consisted of approximately 10–15 weekly individual sessions of PE, a widely disseminated manualized exposure-based intervention for trauma and PTSD (Foa and Rothbaum, 1998). Participants completed an initial (baseline) assessment prior to the initiation of treatment, an assessment immediately following the conclusion of treatment, and a final follow-up assessment 6 months after treatment completion. Follow-up assessment data reported in the current study reflects immediate post-treatment data due to low frequency of 6-month follow-up data.

### Participants

Fourteen (14) veterans with a psychotic spectrum disorder were enrolled in the current study and included in the current analysis plan. These veterans were part of a larger clinical trial for PTSD that included a broader case-mix of SMI diagnoses (Grubaugh et al., 2016). The 14 veterans in the current study consist of the subset of patients from the larger trial that had a diagnosis of a psychotic disorder. Procedures for the current study were identical to that of the larger trial. All veterans had a history of psychiatric hospitalization and impaired psychosocial functioning and generally required assistance with independent living and symptom/medication management as documented within individual patient clinical records and by their disability status. Medication titration was not part of the study, and veterans remained on their current medications and dosing pre- to post-intervention. Diagnoses of a psychotic disorder and PTSD were verified via clinician-administered semi-structured diagnostic interviews (detailed below). Exclusion criteria for study consisted of current substance dependence, current psychiatric hospitalization, or a recent suicide attempt within 2 months of enrollment in the study.

### Assessment Measures

Eligibility for the intervention trial was determined using the following measures:

The Trauma Assessment for Adults (TAA; Resnick, 1996) was used to identify an index trauma for treatment at baseline (i.e., to confirm PTSD criterion A; American Psychiatric Association [APA], 2000). The index trauma was limited to one “type” of traumatic event (i.e., combat or sexual assault) but could include both discrete and/or chronic types of trauma (i.e., single sexual assault, child sexual abuse). In cases where there were multiple events related to the same trauma (i.e., child sexual abuse) a ‘worst’ event was selected if possible and the PTSD assessment was anchored that that ‘worst’ event.

The Clinician-Administered PTSD Scale (CAPS; Blake et al., 2005) assessed the frequency and intensity of current PTSD symptoms using DSM-IV criteria. The CAPS is a well-established measure for determining PTSD diagnoses and possesses strong psychometric properties and diagnostic application (Weathers et al., 2001), and it has been previously utilized to reliably diagnose PTSD in SMI populations (Grubaugh et al., 2011). PTSD diagnoses were confirmed during the baseline assessment with the CAPS using the F1/I2 scoring algorithm (Weathers et al., 1999). Likewise, the CAPS was used as the primary clinical outcome at follow-up.

The Mini-International Neuropsychiatric Interview for DSM-IV (MINI for DSM-IV; Sheehan et al., 1998) is a clinician-administered, semi-structured diagnostic interview that was used to confirm the presence of a psychotic disorder at baseline. Comorbid psychiatric conditions were also identified using the MINI. Veterans who endorsed current alcohol/substance dependence during the MINI were excluded from the intervention trial. The MINI is a well regarded diagnostic tool that demonstrates sufficient diagnostic sensitivity and specificity in comparison to more extensive clinician-administered diagnostic interviews (e.g., SCID; Sheehan et al., 1998).

The PTSD Checklist (PCL; Weathers et al., 1993) is a brief self-report measure of PTSD symptoms based on DSM-IV diagnostic criteria. In the current study, the PCL was administered at each assessment time point and during each treatment session. The PCL has 17 items, and individual item scores are summed to yield a total score ranging from 17 to 85. The PCL demonstrates a high correlation with the CAPS (*r* = 0.93) and sufficient diagnostic efficiency of PTSD (>0.70) within multiple trauma populations (Weathers et al., 1993; Blanchard et al., 1996).

A Reactions to PTSD Research Survey, developed by the study team in previous research, was used at the post-assessment to obtain quantitative ratings of veterans’ reactions to the intervention. The survey used a 10-point Likert scale that assessed six domains: (1) distress, (2) difficulty, and (3) confusion associated with study research procedures; (4) participation satisfaction; (5) perceived value of the research experience; and (6) willingness to participate in comparable research in the future.

A modified Treatment Expectancy (Borkovec and Nau, 1972) form was administered after the third treatment session to assess the subjective treatment outcome expectancies and perceptions of treatment credibility of study participants. Four questions on this measure were selected for use in the current study: (1) patient perception of the treatment rationale; (2) patient confidence in the treatment addressing PTSD; (3) patient willingness to recommend the treatment to others; and (4) patient expectation for treatment success.

### Procedure

Participants were recruited through direct referrals from VA service providers in a specialized mental health clinic, the PTSD Clinical Team (PCT), of a Southeastern VAMC. That is, veterans initially presenting for treatment to this clinic were referred to study personnel if they had a diagnosis of a psychotic disorder in their electronic medical records. Veterans who expressed an interest in receiving treatment through the study were then contacted by study staff to schedule a baseline assessment and determine eligibility. At the time of enrollment, veterans had VA case managers and were on psychotropic medications at the time of enrollment but were not receiving any other psychotherapy at the time. The baseline assessment was conducted by the study team and included a thorough review of the study rationale and procedures followed by informed consent and a battery of instruments. Eligibility was determined by a positive diagnosis of PTSD on the CAPS and a positive diagnosis of a psychotic disorder on the MINI. The study was conducted with full approval from the associated institutional review boards and data collection occurred between January 2008 and December 2012.

### Intervention

Prolonged Exposure was administered, consistent with the PE manual, in 10–15 weekly individual sessions (Foa and Rothbaum, 1998). While PE is implemented in 10 sessions as standardized by the protocol, up to five additional sessions were allowed if clinically warranted, given the novelty of the patient population^1^. Sessions 1 and 2 included psychoeducation, discussion of expectations for therapy, and instruction in diaphragmatic breathing and progressive muscle relaxation. Subsequent sessions consisted of imaginal exposure of the trauma narrative based on the identified index event and *in vivo* exposure exercises based upon a constructed hierarchy of fear provoking stimuli or situations. For patients with multiple traumas of the same theme (i.e., those with multiple combat experiences or child sexual abuse), exposure exercises focused on the ‘worst’ event first and then proceeded to other events as warranted based on habituation to this ‘worst’ event. Treatment sessions lasted approximately 60–90 min and generally included at least 45 min of imaginal exposure.

### Quantitative Statistical Analyses

#### Descriptive Analyses

Statistical assumptions for planned data analyses were evaluated via calculations of demographic and baseline clinical variables descriptive statistics (i.e., measures of central tendency, variability, and frequency distributions). Baseline characteristics for those who completed eight or more sessions of the intervention were compared to the remainder of the sample using Chi-square or Fisher’s Exact Test for dichotomous variables and *t*-tests for continuous variables. Similar comparisons were made between those who attended four or more sessions of the intervention to the remainder of the sample.

#### Feasibility Analyses

The feasibility analyses used the full sample size of 14 veterans with a psychotic spectrum disorder enrolled to receive PE for PTSD. Feasibility outcomes included patients enrolled in the intervention who did not attend any sessions, those who completed only one session, those who completed less than four sessions, those who completed less than eight sessions, and those who completed 11–15 sessions. Assessment items related to treatment expectations and study participation reactions were evaluated with mean/standard deviation calculations.

#### Clinical Outcome Analyses

Clinician-Administered PTSD Scale PTSD analyses consisted of participants who completed at least four treatment sessions (*n* = 10; i.e., received at least two sessions of imaginal exposure). Post-scores were missing for one of the ten subjects who attended at least four sessions, yielding a completer sample of nine participants for CAPS outcome analyses pre- to post-intervention. The full sample size (*n* = 14) was not used because the assumption of missing at random (MAR) was questionable for methods such as multiple imputation, and the sample size was prohibitively small for complex methods for data not missing at random.

The secondary efficacy outcome measure was the PCL evaluated prior to participation in therapy (baseline), after each therapy session during the active intervention phase, and at post-intervention. 95% confidence intervals and the paired *t*-test were used to estimate the magnitude and test statistical significance of change from baseline to post-treatment for CAPS total score and PCL. In a second approach for the PCL, mixed effects modeling (MEM) was used to estimate the slope of scores across the study time period. The trajectory for sessions 1–10 was used for these analyses as this was the standard course of treatment recommended for all patients by the PE manual. Because of the single arm design, analyses of symptom improvement (i.e., efficacy) are considered descriptive and hypothesis generating rather than hypothesis testing.

### Qualitative Data Collection and Analysis

#### Thematic Interview

All participants completed a clinician administered thematic interview at the end of treatment (or when patient was willing, at drop-out) to learn more about their reactions, perceptions, beliefs, preferences, and suggestions for PE. A flexible interview approach was used, providing the patient with topics and subject areas of inquiry but allowing for additional commentary or queries as indicated. Discussions lasted about 30 min and were audiotaped for later transcription and analysis.

#### Data Management/Analysis

Data was coded using a constructivist grounded theory approach (e.g., Charmaz, 2006). First, multiple thorough readings of interview transcriptions by three independent coders was conducted for content analysis. Each independent coder then generated an independent list of thematic categories and subcategories. After the primary coder further developed and ordered the data, the themes were reviewed, refined, and finalized by the group. This analytic approach has been used successfully by the authors with a range of patient and provider samples (e.g., Robins et al., 2005; Frueh et al., 2006a,b, 2012).

## Results

### Descriptive Analyses

See **Table 1** for demographics and baseline severity data on the full sample (*n* = 14) and those included in the completer efficacy analyses (*n* = 10). Comparisons were made between veterans who completed a minimum of eight sessions (i.e., standard course of therapy; *n* = 8) relative to the remainder of the sample (*n* = 6) by age, service era, baseline CAPS total scores, race/ethnicity, marital status, and index trauma. Race/ethnicity, marital status, or index trauma were not statistically different across groups. However, statistically significant group differences emerged by age, service era, and baseline CAPS total scores. More specifically, veterans completing less than eight sessions tended to be younger, *M(SD)* = 38.67(12.50) versus 52.88(11.08), *t* = -2.38, *p* = 0.04; and 50% of those who received less than eight sessions were Operation Enduring Freedom/Operation Iraqi Freedom (OEF/OIF) [χ^2^(3, *n* = 14) = 8.90, *p* = 0.03]. Veterans completing less than eight sessions also had higher baseline CAPS total severity scores relative to the remainder of the sample, *M(SD)* = 87.17(20.12) versus 67.00(15.75), respectively, *t* = 2.11, *p* = 0.05.

**Table 1:** Demographics and baseline symptom severity.

	Full sample *n* = 14	Completer sample *n* = 10
**Age**	*M* = 46.8 *SD* = 12.9	*M* = 52.88 *SD* = 11.75
**Employment**		
Full-time Part-time Unemployed	7% (*n* = 1) 7% (*n* = 1) 86% (*n* = 12)	10% (*n* = 1) 0% 90% (*n* = 13)
**Race**		
African American Caucasian	50% (*n* = 7) 50% (*n* = 7)	
**Relationship status**		
Married/cohabitating Not married/cohabitating	57% (*n* = 8) 43% (*n* = 6)	70% (*n* = 10) 30% (*n* = 4)
**Gender**		
Male	93% (*n* = 13)	100%
**Disability Connection PTSD**		
Yes		42.9% (*n* = 6)
**Service Era**		
Vietnam Post-Vietnam Persian Gulf OEF/OIF	43% (*n* = 6) 14% (*n* = 2) 14% (*n* = 2) 29% (*n* = 4)	
**Index trauma**		
Combat Physical assault Adult sexual assault Serious accident	62.5% (*n* = 8) 12.5% (*n* = 2) 12.5% (*n* = 2) 12.5% (*n* = 2)	
**CAPS**	*M* = 75.64 *SD* = 19.91	*M* = 68.00 *SD* = 18.04
**PCL**	*M* = 64.14 *SD* = 8.31	*M* = 62.00 *SD* = 8.99

*Completers attended four or more sessions and had available CAPS post-data (*n* = 9). Non-completers attended fewer than four session (*n* = 4) or were missing post-data (*n* = 1), yielding a total of *n* = 5 participants.*

Similar comparisons between veterans completing four or more sessions (minimum course of therapy; *n* = 10) relative to the remainder of the sample (*n* = 4), yielded more pronounced differences by CAPS pre-treatment severity scores, *M(SD)* = 94.75(7.80) for those completing less than four sessions versus *M(SD)* = 68.00(18.04) those completing four or more sessions, *t* = 2.81, *p* = 0.01. Age and service era differences became non-significant but demonstrated similar trends, and employment status, race/ethnicity, marital status, and index trauma remained non-significant.

### Feasibility Data

Of the 14 veterans enrolled with a psychotic spectrum disorder, 9 completed a post-treatment assessment/thematic interview (i.e., had complete data). Of the five who did not complete the assessment immediately following the completion of therapy, three dropped out of treatment prior to session three and were not invited for a post-assessment given the recent timing of their initial assessment; the other two declined follow-up. With regard to treatment, 2 veterans attended one session of PE, 10 attended four or more sessions of PE, and 8 attended eight or more sessions of PE, including one (1) veteran who received fifteen sessions (i.e., more than the standard course of treatment)^2^.

#### Treatment Expectancy

Treatment expectancy variables collected *at the end of session 3* suggested that veterans generally found the intervention to be logical, *M(SD)* = 8.14(1.57). Ratings regarding how confident veterans were in a) the treatment addressing PTSD symptoms [*M(SD)* = 5.71(2.56)]; b) likelihood of recommending PE to others [*M(SD)* = 6.43(3.21)]; and c) the intervention successfully decreasing another fear [*M(SD)* = 6.25(3.01)] were all in the moderate to high end.

#### Survey Based Reactions to the Intervention

Reactions to the intervention variables were in the low to moderate range with regard to how (a) distressing [*M(SD)* = 5.33(3.04)]; (b) difficult [*M(SD)* = 2.67(2.40)]; and (c) confusing veterans found the research procedures and intervention [*M(SD)* = 5.22(3.42)]; and in the moderate to high range with regard to how (a) satisfied they were with their participation [*M(SD)* = 8.00(1.50)]; (b) worthwhile they perceived their research participation to be [*M(SD)* = 8.11(1.36)]; and (c) willing they would be to participate in a similar study in the future [*M(SD)* = 7.78(2.73)].

### Clinical Outcomes

**FIGURE 1:**
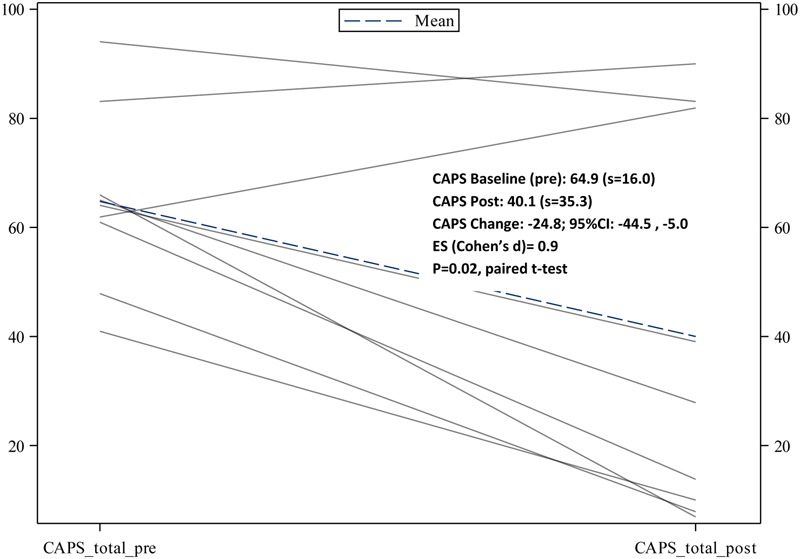
Trajectories of CAPS total scores from baseline to immediate post-treatment.

**FIGURE 2:**
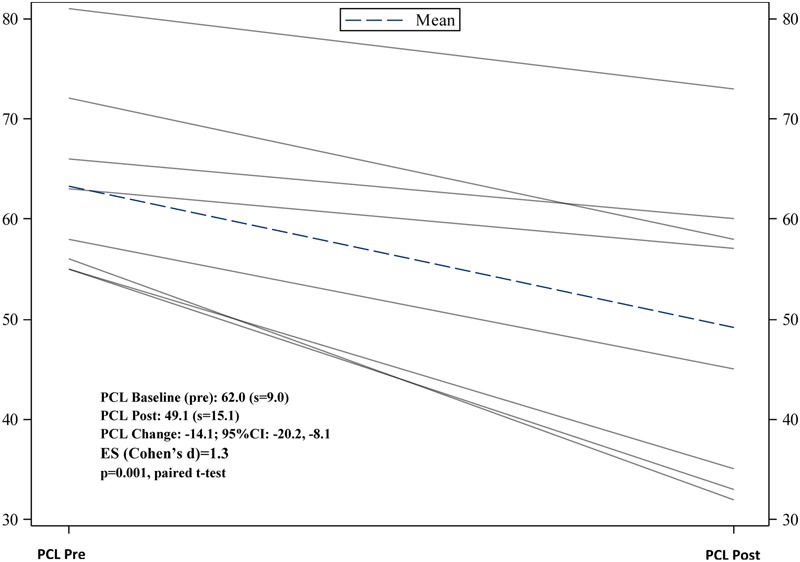
Trajectories of PCL scores from baseline to immediate post-treatment.

**FIGURE 3:**
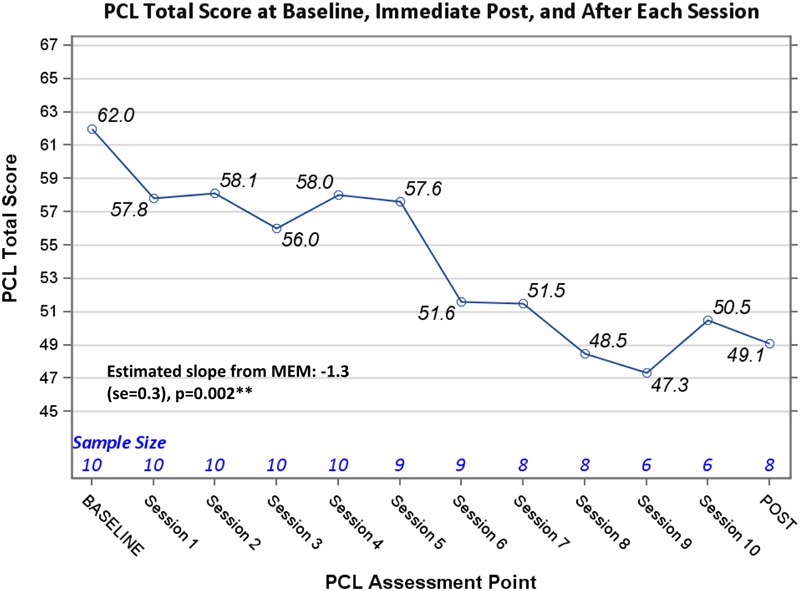
PTSD checklist total score at baseline, immediate post, and after each session.

Completer clinical outcome analyses (*n* = 9) yielded significant pre- to post-changes in CAPS severity scores, -24.8 (95% CI: -44.5, -5.0), *p* = 0.02; as well as significant pre- to post-changes in PCL scores, -14.1(95% CI: -20.0, -8.1), *p* = 0.001. See **Figures 1, 2** for trajectories of CAPS and PCL scores from baseline to post-intervention. With regard to total % change in CAPS scores pre- to post-intervention, 78% of veterans had at least a 10% decrease from baseline to post-intervention, and 55.6% (*n* = 9) had at least a 50% decrease baseline to post-intervention (i.e., responder proportion). Additional analyses examining the trajectory of PCL scores across treatment sessions using MEM provided supportive evidence of a significant decline in PCL means over the treatment trajectory (Estimated slope from MEM: -1.3 (SE = 0.3), *p* = 0.002) (**Figure 3**).

Of the veterans who attended at least eight treatment sessions, six of eight experienced remission of PTSD diagnosis at post-intervention. Additionally, one (1) of the veterans who attended five sessions no longer met criteria for PTSD at post-intervention, yielding a total of seven of nine treatment responders among those who received at least four sessions of the intervention (National Center for PTSD, 2006).

Two veterans remained symptomatic at post-despite receiving at least 10 sessions and another veteran remained symptomatic at post-after completing 8 sessions and dropping out of treatment prematurely. As noted previously, three veterans dropped out of treatment prior to session three, one prior to session four, and another prior to session 6.

### Thematic Interviews

Thematic interviews were designed to learn more about veterans’ reactions, beliefs, preferences, and suggestions for PE. Of the nine thematic interviews available for analysis, six were with veterans who were considered treatment responders (i.e., no longer had PTSD at follow-up and/or experienced a statistically significant decrease in CAPS severity pre- to post-intervention). The other 3 interviews were with veterans who remained symptomatic post-treatment [2 who completed 10 sessions (i.e., the standard course of treatment) and 1 who dropped out of treatment after 8 sessions].

With regard to the content of thematic interviews, five higher order categories were derived based on relative frequency of occurrence in the thematic interview transcriptions:

**Veterans reported long histories of untreated trauma-related symptoms.** Most veterans reported suffering from PTSD for several years, with some index traumas occurring more than 20 years prior to the baseline assessment. Despite this, none of the veterans interviewed reported having received specialized therapy for PTSD in the past. One veteran stated, “*Nobody said anything about PTSD even though I had this problem for a long time, and this is what I kept telling them over and over again, you know*.” Yet another veteran noted, “*Well I’m 52 now and this happened when I was 14 years old and I have never spoken to anyone about it. Till, you know, I did it here…When people used to ask me about it, I would just brush it off, or say I don’t want to talk about it, or that’s the past, I don’t really want to visit it*.”

**Veterans reported being apprehensive at the start of treatment regarding their ability to deal with the difficult emotions and memories that they believed the intervention would solicit.** Veterans, similar to other patient populations enrolled in PE or trauma intensive services, expressed concerns about their ability to manage imaginal exposure sessions (i.e., talking about their trauma in detail) and about the potential adverse consequences of bringing up upsetting/distressing memories. Among those who responded to the intervention, these fears seemed to decrease as treatment progressed. For example, one treatment responder stated, “*Like at first when we started, like it was really difficult to tell the story. It made me upset and made me feel as if I was reliving it as I told it… and as I got to the end, it was almost boring.”* Other treatment responders stated, *“I was a little scared of what it would stir up but I’m happy with the outcome*”; and “*It’s still upsetting, but not like just the whole thing before, it would upset me for like a long time too—you know, for like a couple of hours I would just be in a bad mood, and now not so much, so I think it’s really helped.”* For the three veterans who did not respond to treatment or dropped out prematurely this distress/anxiety seemed to persist.

**Veterans tended to view the treatment and/or treatment team as credible, and this seemed to encourage veterans to “stick with the treatment” despite their anxiety.** One veteran [treatment responder] who reported having a lot of anxiety in the beginning of treatment was asked by the interviewer how he managed his anxiety, and he stated, *“I guess I took a chance and trusted that you guys would help me through it, and that my wife would help me through it.”* Another treatment responder who was not initially confident noted, “*Well at first I thought it’s not going to work. Just the way I can’t really open up that much. But it happened though, something happened and you now, once I got to talking about it and the way I felt afterward, I felt you know real shaky and stuff like that but funny thing about it is, I feel good.”*

Even the three veterans who did not respond to treatment or dropped out prematurely viewed the treatment as logical. One of these veterans stated, “*When I first started right, I felt that the treatment was helping and um I was confident –you know—that I could be helped. Then, as I got deeper into the sessions, I felt that it was bringing things out of me that I didn’t want to deal with. But as far as the treatment, it makes sense. The things that Ms. ^∗∗∗∗^ was showing me made sense and I feel that I, my part of the bargain, you know, I failed because I couldn’t handle it*.”

**Both treatment responders and non-responders reported a number of benefits of the intervention.** Treatment responders tended to report an improved ability to manage thoughts related to the trauma and a greater awareness of how their symptoms were affecting their daily lives. For example, one veteran stated *“It’s okay for me to deal with it now. I realize you know, that it was over there, you know, and it’s not going to happen over here”; and another reported* “*I could think about what happened and not fall to pieces anymore and not have to go to the hospital and stick my head in the sand.*”

Treatment non-responders tended to report being better able to talk about the trauma, a decrease in overall severity of distress [albeit not an elimination of symptoms], and/or having a better ability to manage their trauma-related distress. For example, one veteran stated “*I can talk about it now and before I didn’t want to*”; and yet another veteran who was a treatment non-responder stated, “*On command now I can say stop and gather my thoughts when I’m getting to that point. That helps me you know. The things that I learned I got pretty well embedded in me. So I can always say, well okay, I don’t have to be in control.”*

**Although there was little evidence of relapse or exacerbations in symptoms, some difficulties with the intervention were noted.** Generally speaking, veterans did not report experiencing a worsening of symptoms –either with regard to PTSD or their primary SMI symptoms. Consistent with this, when asked directly whether his symptoms were worse, better, or the same since his enrollment in the study, one veteran [a treatment non-responder] stated, *“They’re about the same. Most days, as long as I don’t think about it, I can live a normal life.”*

Difficulties and/or challenges associated with the intervention generally consisted of initial fears regarding the intensity of the intervention (as noted above) and difficulty managing negative affect outside of sessions. With regard to the latter, one veteran [a treatment non-responder] stated, “*It’s like I said, once I’m here, I can be in here, and talk with her (therapist). She will speak to me and I can go home that day and feel less symptoms, but then another day I’m not seeing her, or something, and I’m back in this rut or whatever, and start not feeling so good, and start thinking about these things, and start hearing things, and seeing things I guess. But I hear them a lot of times too…*. *It just didn’t stick, you know. Just not how I would like it to be.”*

## Discussion

Results of the current study suggest that an exposure-based intervention for PTSD is generally well tolerated and can be therapeutically beneficial among patients with a psychotic spectrum disorder. Quantitative data indicate that seven out of nine veterans experienced remission of PTSD diagnosis at follow-up, and the trajectory of PTSD symptoms over the course of treatment was comparable to general population samples (e.g., Bradley et al., 2005; Cloitre, 2009). Additional data suggest that PE was generally perceived as feasible, logical, and not overly distressing; and veterans typically held favorable expectations with regard to treatment. Qualitative data further suggest that individuals with a psychotic spectrum disorder tend to have similar reactions to PE as other PTSD clinical populations (Foa and Rothbaum, 1998). That is, they have concerns about their ability to manage their distress at the onset of treatment, but this distress typically decreases as they progress through treatment and make gains. Additionally, even treatment non-responders/drop-outs noted some benefits of the intervention, and no veteran experienced a significant exacerbation in PTSD symptoms [or other symptoms] as a result of treatment. Despite misassumptions that trauma-focused therapy may exacerbate symptoms of SMI, no study-related adverse events occurred during the course of the intervention trial. Collectively, these data suggest that individuals with psychotic spectrum disorders do not respond to targeted PTSD intervention in a dramatically different manner than individuals without an SMI.

Notwithstanding a number of positive findings with regard to treatment completers, there were some meaningful differences between those who failed to complete at least four or eight sessions of the intervention and the remainder of the sample. Younger veterans and OEF/OIF veterans were at increased risk to prematurely drop out of the intervention relative to older veterans and other service era cohorts. Given the overlap between age and service era, it is difficult to reach a conclusion regarding the variable dropout of younger veterans and/or OEF/OIF veterans. This result should also be interpreted cautiously, as the non-completer group was small (*n* = 6). Future studies should seek to further understand attrition among OEF/OIF veterans relative to other veteran service era cohorts.

Interestingly, differences in baseline PTSD severity suggested that veterans with the highest levels of PTSD symptoms were at greatest risk for premature treatment dropout in the current analysis. High levels of distress may interfere with treatment adherence. As such, veterans in ‘high’ distress may have benefited from a stronger “buy-in” at the onset of treatment; a shorter time frame between the start of treatment and receipt of imaginal and *in vivo* exposure (i.e., the active elements of PE); motivational interviewing strategies to address ambivalence about treatment; and/or behavioral activation or distress tolerance strategies to manage intense emotions and reduce overall distress. Worth noting again, most veterans dropped out of treatment prior to the start of imaginal exposure, as these patients may not have experienced symptom relief quickly enough to maintain treatment engagement. With regard to treatment non-responders, qualitative data suggest that these individuals continued to have fears about their ability to manage the distress associated with their treatment participation.

Given documented rates of attrition, treatment retention in trauma-focused and/or exposure-based therapies can still be significantly improved. Worth noting, however, the rate of attrition, retention, and treatment completion in the current study is comparable to prior PTSD treatment outcome studies of veterans with combat-related PTSD (Bradley et al., 2005; Cloitre, 2009) and SMI or psychotic spectrum disorders (Rosenberg et al., 2004; Mueser et al., 2007, 2008; Frueh et al., 2009). Additionally, there are no clear guidelines with regard to standard cutoffs for a minimum threshold of dosing for PE. If 3, rather than four sessions, had been used as the cut-off in the current study, our study results would be comparable to a recent study using exposure-based interventions among individuals with a psychotic disorder (van den Berg et al., 2015). With regard to therapeutic dosing, it appears that most veterans responded to a standard course of treatment—and those who remained in treatment tended to get better with some exceptions.

There were some limitations to the current study that require discussion. First and foremost, the sample is small and study data are based on a single arm study design, thus limiting firm conclusions that can be made. Due to the single arm design of our study, inferential analyses of symptom improvement (i.e., efficacy) are considered descriptive and can only provide indications (based on statistically significant *p*-values) that the observed improvement in symptom severity is unlikely the result of chance. That is, the results cannot firmly establish that the treatment produced the changes because unknown intervening events cannot be eliminated as causal factors in the observed improvements in outcomes. Those included in the clinical outcome analyses were significantly older, had less severe PTSD symptoms at baseline, and were predominantly Vietnam era veterans compared to those not included in the analysis sample. Generalization of efficacy results, therefore, is restricted to the population of veterans with SMI having characteristics similar to that of the analysis sample (older veterans with less severe PTSD symptoms). A final limitation concerns the assessment of possible variables that influence successful treatment outcomes. The current study did not include a measure of primary symptoms of psychosis or other well-established predictors of treatment success (i.e., therapist adherence to protocol, rapport, etc.). Addressing such limitations in future studies may provide information regarding how to maximize treatment response and possible additional benefits associated with PTSD treatment in individuals with psychotic spectrum disorders.

The current study addresses important knowledge gaps in the PTSD treatment outcome literature. First, the current study used a mixed methods approach to develop a more comprehensive picture of how individuals with a psychotic spectrum disorder respond to targeted PTSD intervention. Second, the current study represents one of a few examinations of PE, a widely used, researched, and disseminated exposure-based intervention for PTSD, in a patient population with a psychotic spectrum disorder. In this regard the current study findings can be examined within the context of the broader PTSD treatment outcome literature. Finally, study participants reflected a diverse patient population with regards to ethnic/racial identification [i.e., the majority of the sample (*n* = 7; 50%) identified as minority].

Altogether, the current study findings are promising and serve to mitigate both provider and patient beliefs that individuals with psychotic spectrum disorders such as schizophrenia cannot benefit from intense trauma focused treatment. Additional data along this theme would further disseminate the use of empirically supported PTSD interventions in this patient population. Such efforts would undoubtedly facilitate the establishment of an evidence-based standard of care and the incorporation of trauma-focused interventions into the psychosocial rehabilitation conceptualization for this patient population.

## Ethics Statement

Medical University of SC Institutional Review Board and Ralph H. Johnson VAMC Research and Development Office Participants were recruited via direct referrals from VA providers to a PTSD Clinical Team (PCT) of a Southeastern VAMC. All interested individuals were given a description of the study over the phone by project staff (or in-person if a patient stopped by without an appointment); and if willing, were scheduled for a formal baseline assessment. The baseline assessment included a detailed explanation of the study and informed consent. The study was conducted between January of 2008 and December of 2012 with full approval from appropriate institutional review boards. All participants completed a brief multiple choice quiz to ensure their understanding of the study procedures.

## Author Contributions

AG oversaw manuscript development, participated in the analysis and interpretation of data, and took the lead in drafting the majority of the manuscript. CE, RK, WB, and KV contributed to the analysis and interpretation of the data; RK drafted portions of the quantitative analysis section; WB, KV, and CE contributed to the write-up of the qualitative analysis section. All authors contributed feedback for the Discussion section.

## Conflict of Interest Statement

The authors declare that the research was conducted in the absence of any commercial or financial relationships that could be construed as a potential conflict of interest.

## Footnotes

1 Veterans were offered additional sessions by their therapist if they reported significant symptoms on the PTSD Symptom Checklist (PCL) toward the end of the standard course of treatment (i.e., by session 7 or 8). Only one Veteran in the current sample was offered and received additional sessions.

2 Of the 6 veterans who did not complete the standard course of treatment (i.e., 8 or more sessions), 2 cited transportation problems as their reason for drop-out; 2 did not respond to follow-up attempts, 1 was a treatment responder who no longer wanted treatment due to symptom amelioration; and 1 stated he was no longer interested in participating in the study.
